# Depression among people with type 2 diabetes mellitus, US National Health and Nutrition Examination Survey (NHANES), 2005–2012

**DOI:** 10.1186/s12888-016-0800-2

**Published:** 2016-04-05

**Authors:** Yiting Wang, Janice M. S. Lopez, Susan C. Bolge, Vivienne J. Zhu, Paul E. Stang

**Affiliations:** Janssen Research & Development, LLC, 1125 Trenton Harbourton Road, Titusville, NJ 08560 USA; Janssen Scientific Affairs, LLC, Raritan, 1000 US Route 202, Raritan, NJ 08869 USA; Department of Public Health Sciences, Medical University of South Carolina, 135 Cannon Street Suite 303, Charleston, SC 29425 USA

**Keywords:** Depression, Patient Health Questionnaire (PHQ-9), Type 2 diabetes mellitus (T2DM)

## Abstract

**Background:**

Depression in people with diabetes can result in increased risk for diabetes-related complications. The prevalence of depression has been estimated to be 17.6 % in people with type 2 diabetes mellitus (T2DM), based on studies published between 1980 and 2005. There is a lack of more recent estimates of depression prevalence among the US general T2DM population.

**Methods:**

The present study used the US National Health and Nutrition Examination Survey (NHANES) 2005–2012 data to provide an updated, population-based estimate for the prevalence of depression in people with T2DM. NHANES is a cross-sectional survey of a nationally representative sample of the civilian, non-institutionalized US population. Starting from 2005, the Patient Health Questionnaire (PHQ-9) was included to measure signs and symptoms of depression. We defined PHQ-9 total scores ≥ 10 as clinically *relevant* depression (CRD), and ≥ 15 as clinically *significant* depression (CSD). Self-reported current antidepressant use was also combined to estimate overall burden of depression. Predictors of CRD and CSD were investigated using survey logistic regression models.

**Results:**

A total of 2182 participants with T2DM were identified. The overall prevalence of CRD and CSD among people with T2DM is 10.6 % (95 % confidence interval (CI) 8.9–12.2 %), and 4.2 % (95 % CI 3.4–5.1 %), respectively. The combined burden of depressive symptoms and antidepressants may be as high as 25.4 % (95 % CI 23.0–27.9 %). Significant predictors of CRD include age (younger than 65), sex (women), income (lower than 130 % of poverty level), education (below college), smoking (current or former smoker), body mass index (≥30 kg/m^2^), sleep problems, hospitalization in the past year, and total cholesterol (≥200 mg/dl). Significant predictors of CSD also include physical activity (below guideline) and cardiovascular diseases.

**Conclusions:**

The prevalence of CRD and CSD among people with T2DM in the US may be lower than in earlier studies, however, the burden of depression remains high. Further research with longitudinal follow-up for depression in people with T2DM is needed to understand real world effectiveness of depression management.

**Electronic supplementary material:**

The online version of this article (doi:10.1186/s12888-016-0800-2) contains supplementary material, which is available to authorized users.

## Background

Depression can increase the risk of diabetes-related complications in people with diabetes due to poor self-care, reduced treatment adherence, and poor glycemic control [1]. The management of comorbid depression and diabetes through integrated and collaborative care has been shown to improve medication adherence, glycemic control [2], and depression outcomes [3]. Routine screening of depression in adults with diabetes is recommended by the American Association of Clinical Endocrinologists (AACE) [4]. Integrating depression screening with cost-effective treatment strategies has also been recommended for the general primary care setting [5]. Yet, it has been suggested that few diabetes clinics provide mental health screening or integrate mental/behavioral health services in diabetes clinical care, and even the use of brief paper-and-pencil self-report measures remains rare [6].

There is extensive evidence that depression is a common comorbidity in people with diabetes [7]. One meta-analysis reported an overall depression prevalence of 27 % in people with type 2 diabetes mellitus (T2DM) based on 15 studies published before January 2000 [8]. A later meta-analysis reported an overall depression prevalence of 17.6 % in people with T2DM compared with 9.8 % in those without T2DM, based on 10 studies published between January 1980 and May 2005 with a total of 51,331 people across multiple countries, including the US [9]. There is only a limited number of large, population-based studies that estimated depression prevalence for people with T2DM. The Kaiser Permanente Diabetes Registry reported a prevalence of diagnosed depression (based on diagnosis codes in medical records) to be 17.9 % in T2DM in 1999 [10]. A study of the 2006 Preventive Behavioral Risk Factor Surveillance System (PBRFSS) (a cross-sectional, random-sample, telephone survey) reported depression prevalence of 24 % in people with T2DM using insulin, and 17.3 % in people with T2DM not using insulin (depression was defined as total score ≥10 on the Patient Health Questionnaire, PHQ-8, administered in 36 states and territories in 2006) [11]. There is a lack of more recent, US national-level estimates of depression prevalence among a representative sample of the T2DM population.

The present study used the US National Health and Nutrition Examination Survey (NHANES) 2005–2012 data to estimate the prevalence of depression, and to explore potential predictors of clinically *relevant* and clinically *significant* depression (defined below) in a nationally representative sample that includes people with T2DM.

## Methods

The US National Health and Nutrition Examination Survey (NHANES) is a cross-sectional survey of a nationally representative sample of the civilian, non-institutionalized US population, designed to monitor the nation’s health and nutrition status [12]. NHANES uses a complex, multistage, probability sampling design to select participants, and continuous data collection has been conducted in 2-year cycles since 1999 [12]. During each 2-year cycle, about 30 selected counties (i.e., the primary sampling units) were visited out of approximately 3,000 counties in the US. Within selected segments of these counties, letters explaining NHANES were sent to selected households before trained interviewers visited and obtained informed consent from individuals for participation. Each 2-year survey cycle has a combined sample of roughly 10,000 persons and is representative of the civilian, non-institutionalized US population. Overall interview response rate was roughly 80 % (see Additional file 1).

The survey comprises interviews conducted in participants’ homes and standardized physical examinations in mobile examination centers (MECs), including laboratory tests. Topics covered by the survey questionnaires include demographics (e.g., age, sex, race/ethnicity, family income-to-poverty ratio [13], education, and marital status), general health (e.g., hospitalization in the past year, weight change history, hours of sleep, sleep problems), behavior and lifestyle variables (e.g., smoking, alcohol, physical activities, substance use), disease history (e.g., whether they have been diagnosed by health professionals to have diabetes, hypertension, cancer, cardiovascular diseases, liver disease). NHANES participants reported prescription medication use during a one-month period prior to the survey date, and were asked to show the interviewer their medication containers during the household interview. Lexicon Plus®, a proprietary database of Cerner Multum, Inc. [14] was used in NHANES to assist with medication data collection, data editing and release. The Lexicon Plus® is a comprehensive database of all prescription and some nonprescription drug products available in the US drug market. We identified antidepressants using the Lexicon Plus® therapeutic classification (first-level category “PSYCHOTHERAPEUTIC AGENTS”, second-level category “ANTIDEPRESSANTS”), which included selective serotonin reuptake inhibitors [SSRI], monoamine oxidase inhibitors [MAOI], tricyclic antidepressants [TCA], serotonin-norepinephrine reuptake inhibitors [SNRI], phenylpiperazine and miscellaneous antidepressants [15]. About 85 % of the self-reported use of antidepressants had a record of “prescription container seen by the interviewer”.

Starting from 2005, NHANES has included the Patient Health Questionnaire (PHQ-9) [16, 17] to measure signs and symptoms of depression. The PHQ-9 and some other computer-assisted personal interviews, and anthropometrics (e.g., body weight, height, waist-to-height ratio) as well as bio-specimens are taken in the MEC according to standardized protocols. The PHQ-9 is a 9-item depression screening instrument that asks about the frequency of symptoms of depression over the past 2 weeks [16, 17]. Total PHQ-9 score ranges from 0 to 27 and are categorized as “none or minimum” (0–4), “mild” (5–9), “moderate” (10–14), “moderately severe” (15–19), and “severe” (20–27) for depression severity. In this study, we combined scores 10–27 (“moderately severe or severe”) to improve precision of estimates. We defined PHQ-9 total scores ≥ 10 as clinically *relevant* depression (CRD) [18], which is consistent with depression process/outcome performance measures recommended by the National Quality Forum [19]. PHQ-9 score ≥10 has shown a sensitivity of 88 % and a specificity of 88 % for major depression [17]. In addition, those with PHQ-9 scores ≥ 15 (a subset of those with PHQ-9 scores ≥10) were categorized as clinically *significant* depression (CSD), which suggests the presence of major depression for which active treatment with pharmacotherapy and/or psychotherapy is recommended [17]. Participants reporting current antidepressant use were also included as prevalent cases in the estimation of overall burden of depressive symptoms because this may imply CRD and/or CSD. Because some antidepressants are also indicated for other psychiatric symptoms (e.g., anxiety), and because the reasons for taking antidepressants were not available from NHANES, we conducted sensitivity analyses of various combinations of PHQ-9 score, antidepressant use and consultation with mental health professionals. We also analyzed question 9 of the PHQ-9 questionnaire which asks about suicidal ideation. The specificity of the PHQ-9 suicide screening item was 0.84 and sensitivity was 0.69 in a study of 166 people from 2 primary care clinics in the US, which suggested it may be useful in primary care practice to identify individuals at risk for suicide [20]. In addition to the PHQ-9 questions, NHANES also assesses the difficulty caused by the depressive symptoms with work, taking care of things at home, or getting along with people, using a scale of ‘not at all’ to extremely difficult.

We identified people with T2DM using the following criteria [21, 22]: self-reported diagnosis of diabetes or “sugar diabetes” at age ≥ 30 years, not initiating insulin therapy within 1 year of diabetes diagnosis, and not pregnant at the time of interview/examination. Diabetes-related variables such as age at diagnosis, self-monitoring of blood glucose, diabetes complications and antihyperglycemic medications were based on NHANES diabetes questionnaire data, lab/exam variables such as glycohemoglobin (HbA1c), cholesterol, and blood pressure were based on NHANES MEC data.

Analyses were weighted, and accounted for the stratified, multistage probability sampling design of NHANES and survey nonresponse. Logistic regression models were used to select predictors of CRD and CSD in T2DM from potential candidate variables [9, 23], including demographics, general health, behavior and lifestyle, disease history, lab and prescription medications. First, univariable association of all the potential predictors were evaluated (i.e., entered into the survey logistic regression models one at a time), and those with statistically significant association with CRD and/or CSD were then entered together into a multivariable regression model. The final multivariable model retained all variables that remained statistically significant. Statistical significance was assessed by two-sided *P* values of < 0.05, with no adjustments for multiple testing. Odds ratio (OR) and 95 % confidence intervals (CI) were estimated and presented for predictors selected in the final models. We did not use stepwise automated model selection because the effective degrees of freedom are bounded by the number of clusters (i.e., primary sampling units in NHANES), which makes such methods problematic [24–26]. We also compared analyses that excluded missing PHQ-9 data with analyses that combined missing PHQ-9 data with the non-CRD and non-CSD categories.

We also evaluated whether there was a temporal trend in the prevalence of depressive symptoms or antidepressant use over the 8 years (i.e., 4 NHANES survey cycles). This was done by fitting NHANES survey cycle as a continuous variable in the survey regression models. All analyses were performed using SAS (SAS Institute Inc., Cary, NC, USA), version 9.2.

## Results

There were a total of 40,790 participants in the NHANES 2005–2012 (which represents the total US civilian, non-institutionalized population of 299.3 million people). Among these, a total of 2,648 respondents reported that they had been “told by a doctor or health professional” that they had “diabetes or sugar diabetes” (this sample represents 18.8 million people with diagnosed diabetes). From the 2,648 respondents, we identified 2,182 participants with T2DM by our definition and who had data for both the interview and physical examination. Among the 2,182 respondents with T2DM, 235 (10.8 %) had missing data on one or more items of the PHQ-9 questionnaire, including 37 (1.7 %) taking antidepressants. We compared analyses that excluded missing PHQ-9 data with analyses that combined the missing data with the non-CRD and non-CSD categories. Results were similar and the latter are presented.

Tables 1 and 2 describe the general characteristics of people with T2DM according to clinically *relevant* depression (CRD, defined by PHQ-9 total score > =10) and clinically *significant* depression (CSD, defined by PHQ-9 total score > =15). (See Additional file 2 for the descriptions according to the four PHQ-9 categories). Overall, the categories for more severe depression have a higher proportion of T2DM subjects who were younger, women, minority ethnicities (i.e., not non-Hispanic White), single or living alone, less physically active, having lower income, lower education level, sleep problems, cardiovascular diseases, cancer, liver and kidney diseases.

**Table 1:** Characteristics of people with T2DM by *clinically relevant depression*, NHANES 2005–2012

	Non-CRD	CRD	Overall
N, participants (%)	1926 (88.3)	256 (11.7)	2182 (100.0)
Frequency, weighted^a^ (%)	13,520,141(89.4)	1,597,475(10.6)	15,117,616 (100.0)
Age group, %			
30–49 years	15.4	27.5	16.6
50–64 years	38.1	43.7	38.7
65–74 years	26.7	18.8	25.8
≥ 75 years	19.9	10.0	18.8
Sex, %			
Male	50.8	30.7	48.7
Female	49.2	69.3	51.3
Race, %			
Non-Hispanic white	61.1	54.6	60.4
Non-Hispanic black	17.3	21.6	17.8
Mexican and other Hispanic	12.9	19.9	13.6
Other	8.7	3.9	8.2
Ratio of family income to poverty level ≤ 1.3, %	20.8	52.1	24.1
Marital status, single or living alone, %	36.3	54.5	38.3
Education, %			
College or above	47.2	31.6	45.5
High school graduate	25.3	22.6	25.0
Below high school	27.5	45.8	29.4
Smoking			
Non-smoker	51.0	39.3	49.8
Past	36.0	28.5	35.2
Current	13.0	32.2	15.0
Alcohol			
Non-drinker	42.4	49.4	43.2
Above moderate level	16.0	21.2	16.6
Moderate drinking	41.5	29.4	40.2
Marijuana, ever use, %	16.5	28.9	17.8
Physical activity level met guidelines,^b^ %	43.1	26.7	41.4
Cardiovascular diseases	28.0	36.4	28.8
Diabetic retinopathy	18.0	25.4	18.8
Hypertension	81.7	81.5	81.7
Liver diseases	4.1	7.7	4.5
Cancer	17.4	18.5	17.5
Weak/failing kidneys^c^	7.3	12.2	7.8
Overnight hospitalization in the past year, %	20.5	44.0	23.0
My health in general is, %			
Excellent, very good	16.3	2.5	14.8
Good/Fair	69.6	65.2	69.1
Poor	6.5	32.4	9.2
Missing	7.7	0.0	6.9
Hours of sleep per day, %			
< =5 h	16.2	38.5	18.6
6-8 h	74.1	50.7	71.6
> =9 h	9.1	9.9	9.2
Missing	0.5	1.0	0.6
Self-reported having trouble sleep, %	33.8	65.2	37.1
Told by doctor having sleep disorders, %	15.7	29.9	17.2
Age at diabetes diagnosis, years			
Mean (s.e.)	52.6 (0.4)	48.9 (0.9)	52.2 (0.4)
Categories, %			
< 50	41.6	55.6	43.0
50–69	47.9	38.3	46.8
> =70	9.8	4.8	9.3
Missing	0.8	1.3	0.8
Duration of diabetes			
Mean (s.e.)	10.0 (0.2)	8.9 (0.7)	9.9 (0.2)
Categories, %			
< 5 years	30.8	28.3	30.5
5–9 years	23.9	29.9	24.5
> =10 years	44.5	40.6	44.1
Missing	0.8	1.3	0.8
HbA1c, %			
Mean (s.e.)	7.3 (0.1)	7.4 (0.2)	7.3 (0.1)
Categories, %			
< 6.5 %	34.8	34.0	34.7
6.5- < 7.5 %	29.1	25.3	28.7
7.5–8.9 %	18.9	17.9	18.8
> =9 %	13.0	13.4	13.0
Missing	4.2	9.5	4.7
Total cholesterol (mg/dl)			
Mean (s.e.)	182.0 (1.6)	193.1 (3.7)	183.1 (1.5)
Categories			
> 0 and <200	65.8	49.8	64.1
> =200	27.9	39.9	29.2
Missing	6.3	10.3	6.7
LDL cholesterol(mg/dl)			
Mean (s.e.)	98.1 (1.4)	108.4 (4.1)	99.0 (1.4)
Categories			
> 0 and <100	52.7	44.9	52.0
> =100	39.4	48.5	40.2
Missing	7.9	6.6	7.8
HDL cholesterol (mg/dl)			
Male and female, mean (s.e.)	47.8 (0.4)	45.8 (1.1)	47.6 (0.4)
Male, mean (s.e.)	44.2 (0.5)	41.2 (2.0)	44.0 (0.5)
Female, mean (s.e.)	51.7 (0.7)	48.0 (1.2)	51.2 (0.7)
Categories, %			
Male > 40 and female > 50	49.7	36.9	48.4
Male < =40 and female < =50	43.9	52.8	44.9
Missing	6.3	10.3	6.7
Triglycerides (mg/dl)			
Mean (s.e.)	176.3 (9.8)	180.1 (14.0)	176.6 (9.4)
Categories			
> 0 and <150	55.6	46.3	54.8
> =150	41.8	50.4	42.6
Missing	2.5	3.3	2.6
Blood pressure (BP), mmHg			
SBP, mean (s.e.)	130.6 (0.6)	128.6 (1.4)	130.4 (0.5)
DBP, mean (s.e)	68.4 (0.5)	70.7 (1.0)	68.6 (0.4)
SBP < 140, DBP < 90	67.1	72.3	67.5
Missing	4.9	2.3	4.6
Medications			
Insulin, %	27.0	24.7	26.8
Sulfonylureas, %	35.8	27.0	34.8
THIAZOLIDINEDIONES, %	17.5	14.3	17.1
Meglitinides, %	2.0	0.2	1.8
DPP-4 INHIBITORS, %	7.6	1.4	6.9
GLP-1 AGONISTS, %	1.5	1.8	1.5
Metformin, %	52.2	59.7	53.0
Any antihyperglycemic agents	88.1	87.3	88.0
Antihypertensives, %	76.7	76.0	76.6
Statins, %	54.3	52.0	54.1
Body mass index, kg/m^2^			
Mean (s.e.)	32.5 (0.3)	35.5 (0.6)	32.8 (0.2)
Categories, %			
< 25	13.9	8.2	13.3
25- < 30	26.7	11.0	25.0
30- < 35	27.4	37.1	28.5
35+	31.9	43.7	33.2
Missing	0.1	0.0	0.1
Waist-to-height ratio, mean (s.e.)	0.659 (0.004)	0.699 (0.007)	0.663 (0.003)
Categories, %			
0.3- < 0.5	2.4	1.4	2.3
0.5- < 0.7	62.6	48.4	61.1
≥ 0.7	26.8	40.2	28.2
Missing	8.3	10.0	8.5

Values are % unless stated otherwise, percentages may not add up exactly to 100 due to rounding

*CRD* clinically relevant depression, defined by PHQ-9 score ≥ 10; S.e. = standard error

^a^There were 466 participants (=2,648-2,182) who reported having diagnosed diabetes but were not included in this analysis (due to presumed type 1 diabetes or non-participation in the MEC exams), representing 3,653,851 (=18,771,467-15,117,616) people with diabetes in the US civilian, non-institutionalized population

^b^Physical activity level evaluated according to the 2008 Physical Activity Guidelines for Americans [36]

^c^A “yes” answer to “Have you ever been told by a doctor or other health professional that you have weak or failing kidneys (excluding kidney stones, bladder infections, or incontinence)?”

**Table 2:** Characteristics of people with T2DM by *clinically significant depression*, NHANES 2005–2012

	Non-CSD	CSD	Overall
N, participants	1926 (88.3)	256 (11.7)	2182
Frequency, weighted^a^ (%)	13,520,141(89.4)	1,597,475(10.6)	15,117,616 (100.0)
Age group, %			
30–49 years	16.3	25.4	16.6
50–64 years	38.2	50.3	38.7
65–74 years	26.3	15.4	25.8
≥ 75 years	19.3	8.9	18.8
Sex, %			
Male	49.5	29.4	48.7
Female	50.5	70.6	51.3
Race, %			
Non-Hispanic white	60.8	50.6	60.4
Non-Hispanic black	17.6	23.4	17.8
Mexican and other Hispanic	13.3	21.8	13.6
Other	8.3	4.2	8.2
Ratio of family income to poverty level ≤ 1.3, %	22.4	62.9	24.1
Marital status, single or living alone, %	37.6	53.3	38.3
Education, %			
College or above	46.5	23.7	45.5
High school graduate	25.3	19.5	25.0
Below high school	28.2	56.8	29.4
Smoking			
Non-smoker	50.2	39.1	49.8
Past	35.7	24.7	35.2
Current	14.1	36.1	15.0
Alcohol			
Non-drinker	42.5	58.9	43.2
Above moderate level	16.4	20.3	16.6
Moderate drinking	41.1	20.8	40.2
Marijuana, ever use, %	17.3	29.3	17.8
Physical activity level met guidelines,^b^ %	42.5	16.9	41.4
Cardiovascular diseases	28.3	41.9	28.8
Diabetic retinopathy	18.8	18.6	18.8
Hypertension	81.8	79.1	81.7
Liver diseases	4.2	11.9	4.5
Cancer	17.3	23.8	17.5
Weak/failing kidneys^c^	7.6	13.1	7.8
Overnight hospitalization in the past year, %	22.1	42.5	23.0
My health in general is, %			
Excellent, very good	15.4	1.2	14.8
Good/Fair	69.4	61.7	69.1
Poor	8.0	37.1	9.2
Missing	7.2	0.0	6.9
Hours of sleep per day, %			
< =5 h	17.5	43.5	18.6
6–8 h	73.0	40.2	71.6
> =9 h	8.9	15.6	9.2
Missing	0.6	0.7	0.6
Self-reported having trouble sleep, %	35.7	69.2	37.1
Told by doctor having sleep disorders, %	16.3	38.2	17.2
Age at diabetes diagnosis, years			
Mean (s.e.)	52.4 (0.4)	48.4 (1.3)	52.2 (0.4)
Categories, %			
< 50	42.5	56.1	43.0
50–69	47.3	37.2	46.8
> =70	9.5	4.8	9.3
Missing	0.8	1.9	0.8
Duration of diabetes			
Mean (s.e.)	9.9 (0.2)	9.6 (1.0)	9.9 (0.2)
Categories, %			
< 5 years	30.9	21.1	30.5
5–9 years	24.3	29.3	24.5
> =10 years	43.9	47.6	44.1
Missing	0.8	1.9	0.8
HbA1c, %			
Mean (s.e.)	7.3 (0.1)	7.4 (0.2)	7.3 (0.1)
Categories, %			
< 6.5 %	34.9	31.7	34.7
6.5- < 7.5 %	28.7	28.7	28.7
7.5–8.9 %	19.1	11.9	18.8
> =9 %	12.9	15.0	13.0
Missing	4.4	12.8	4.7
Total cholesterol (mg/dl)			
Mean (s.e.)	182.6 (1.6)	195.3 (5.3)	183.1 (1.5)
Categories			
> 0 and <200	65.1	42.4	64.1
> =200	28.5	43.4	29.2
Missing	6.4	14.2	6.7
LDL cholesterol(mg/dl)			
Mean (s.e.)	98.5 (1.4)	112.9 (6.0)	99.0 (1.4)
Categories			
> 0 and <100	52.5	38.9	52.0
> =100	39.7	54.3	40.2
Missing	7.8	6.8	7.8
HDL cholesterol (mg/dl)			
Male and female, mean (s.e.)	47.7 (0.4)	45.1 (1.7)	47.6 (0.4)
Male, mean (s.e.)	44.1 (0.5)	38.7 (2.7)	44.0 (0.5)
Female, mean (s.e.)	51.3 (0.7)	48.0 (1.6)	51.2 (0.7)
Categories, %			
Male > 40 and female > 50	49.1	31.7	48.4
Male < =40 and female < =50	44.5	54.0	44.9
Missing	6.4	14.2	6.7
Triglycerides (mg/dl)			
Mean (s.e.)	177.1 (9.7)	162.4 (14.7)	176.6 (9.4)
Categories			
> 0 and <150	55.1	47.6	54.8
> =150	42.5	45.5	42.6
Missing	2.4	6.8	2.6
Blood pressure (BP), mmHg			
SBP, mean (s.e.)	130.5 (0.6)	126.6 (2.1)	130.4 (0.5)
DBP, mean (s.e)	68.6 (0.4)	68.8 (1.9)	68.6 (0.4)
SBP < 140, DBP < 90	67.7	64.3	67.5
Missing	4.6	4.5	4.6
Medications			
Insulin, %	26.6	30.3	26.8
Sulfonylureas, %	35.4	22.1	34.8
THIAZOLIDINEDIONES, %	17.5	9.9	17.1
Meglitinides, %	1.8	0.4	1.8
DPP-4 INHIBITORS, %	7.3	0.0	6.9
GLP-1 AGONISTS, %	1.5	2.9	1.5
Metformin, %	53.0	53.7	53.0
Any antihyperglycemic agents	88.3	81.6	88.0
Antihypertensives, %	76.8	73.2	76.6
Statins, %	54.5	45.2	54.1
Body mass index, kg/m^2^			
Mean (s.e.)	32.7 (0.2)	34.4 (0.7)	32.8 (0.2)
Categories, %			
< 25	13.5	9.6	13.3
25- < 30	25.5	13.5	25.0
30- < 35	28.1	35.9	28.5
35+	32.8	41.0	33.2
Missing	0.1	0.0	0.1
Waist-to-height ratio, mean (s.e.)	0.662 (0.003)	0.691 (0.010)	0.663 (0.003)
Categories, %			
0.3- < 0.5	2.3	1.8	2.3
0.5- < 0.7	61.7	46.5	61.1
≥ 0.7	27.8	37.4	28.2
Missing	8.2	14.3	8.5

Values are % unless stated otherwise, percentages may not add up exactly to 100 due to rounding. *CSD* Clinically significant depression, defined by PHQ-9 score ≥ 15; S.e. = standard error

^a^There were 466 participants (=2,648-2,182) who reported having diagnosed diabetes but were not included in this analysis (due to presumed type 1 diabetes or non-participation in the MEC exams), representing 3,653,851 (=18,771,467-15,117,616) people with diabetes in the US civilian, non-institutionalized population

^b^Physical activity level evaluated according to the 2008 Physical Activity Guidelines for Americans [42]

^c^A “yes” answer to “Have you ever been told by a doctor or other health professional that you have weak or failing kidneys (excluding kidney stones, bladder infections, or incontinence)?”

**Fig. 1:**
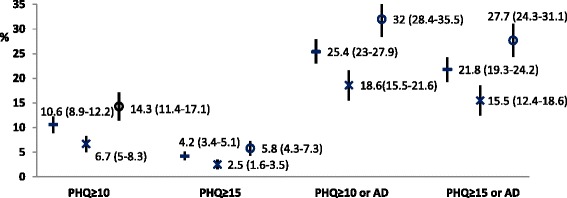
Prevalence of CRD, CSD and antidepressant treatment in T2DM, NHANES 2005–2012. Legends: prevalence in men and women with T2DM overall shown by “-”, in men with T2DM by “x”, and in women with T2DM by “o”, respectively. Vertical lines extend from lower to upper 95 % confidence limits of the corresponding prevalence estimates. CRD, *clinically relevant depression*, defined by PHQ score ≥10; CSD, *clinically significant depression*, defined by PHQ score ≥15; AD, currently taking antidepressants

Figure 1 plots prevalence estimates of CRD and CSD. Between 2005 and 2012, the prevalence of CRD among people with T2DM is 10.6 % overall (95 % CI 8.9–12.2 %), and the prevalence is higher in women than in men. Similarly, the overall T2DM population prevalence of CSD is 4.2 % (95 % CI 3.4–5.1 %). However, when antidepressant medication is taken into consideration, the combined burden of depression may be as high as 25.4 % overall (95 % CI 23.0–27.9 %). In a sensitivity analysis that removed some common antidepressants (see Table 3, last row) approved for other psychiatric indications (mainly anxiety and related disorders) in the US, the overall burden of CRD and/or antidepressants was 16.7 % (95 % CI 14.6–18.9 %), and the overall burden of CSD and/or antidepressants was 10.8 % (95 % CI 8.9–12.7 % in people with T2DM. In women, the corresponding prevalence were 20.5 % (95 % CI 16.7–24.3 %), and 12.3 % (95 % CI 9.2–15.4 %); in men, the corresponding prevalence were 12.7 % (95 % CI 10.5–15.0 %) and 9.2 % (95 % CI 7.3–11.1 %), respectively. There was a statistically significant trend in the prevalence of CRD and CSD, which increased early on and then stabilized from the 2009–2010 to the 2011–2012 period (Additional file 3). The increasing trend was noted at higher PHQ-9 scores (i.e., PHQ-9 score ≥ 15 and PHQ-9 score ≥ 20). No significant trend was detected in the prevalence of antidepressant use, although there appeared to be a drop in antidepressant use from the 2005–2006 to the 2007–2008 period. We did not find any notable differences in missing data pattern for PHQ-9 data across these years.

**Table 3:** Mental health status by severity of depressive symptoms

Characteristic	PHQ-9 depression score					Overall
	0–4	5–9	10–14	15–27	Missing	
	None/minimal	Mild	Moderate	Moderately severe/Severe		
Difficulty with work, life, people due to depressive symptoms, %						
Not at all	48.0	56.5	37.6	22.3	2.8	43.4
Somewhat	9.5	39.6	42.1	22.2	1.4	16.5
Very	0.8	2.8	17.0	31.6	0.4	3.4
Extreme	0.2	1.1	3.3	23.2	0.5	1.5
Missing	41.6	0.0	0.0	0.7	94.9	35.2
Suicidal ideation, %						
Not at all	99.5	90.4	83.9	55.6	6.2	86.3
Several days	0.5	8.9	14.4	13.9	0.7	3.4
More than half the days	0	0.3	0.8	11.1	0.2	0.6
Nearly every day	0	0.4	0.9	19.4	0	0.9
Missing	0	0	0	0	92.9	8.8
# of days mental health not good during the past 30 days						
Mean (s.e.)	2.0 (0.2)	6.9 (0.6)	13.4 (1.3)	19.5 (1.3)	5.4 (1.4)	4.6 (0.3)
Categories, %						
< 15 days	94.4	80.5	53.6	30.5	21.4	79.9
> =15 and < =30	5.5	19.4	46.4	68.2	4.0	13.0
Missing	0.1	0.1	0.0	1.3	74.6	7.2
# of days physical health not good during the past 30 days						
Mean (s.e.)	3.9 (0.3)	8.7 (0.8)	16.1 (1.3)	18.9 (1.3)	6.3 (1.4)	6.4 (0.3)
Categories, %						
< 15 days	89.0	73.9	46.8	36.6	20.8	75.1
> =15 and < =30	10.8	26.1	53.2	62.9	5.4	17.8
Missing	0.2	0.1	0.0	0.5	73.8	7.1
# of days during the past 30 days, poor physical/mental health kept from doing usual activities, such as self-care, work, school or recreation						
Mean (set 77, 99 = .)	1.8 (0.2)	5.2 (0.7)	11.8 (1.4)	15.4 (1.7)	6.7 (1.8)	3.9 (0.3)
Categories, %						
< 15 days	94.9	84.0	63.2	43.0	19.8	81.7
> =15 and < =30	4.9	15.9	36.8	55.3	6.4	11.1
Missing	0.2	0.1	0.0	1.7	73.8	7.2
Seen mental health professional past 12 months, %	9.1	7.4	12.6	14.0	8.4	9.2
Antidepressants, %	14.1	24.9	42.2	49.4	18.1	19.6
SSRI, %	8.9	15.6	24.2	31.5	11.2	12.2
SSNRI, %	2.1	2.8	10.7	9.0	4.7	3.3
Duloxetine, %	1.0	0.3	1.1	0.0	0.9	0.8
TCA, %	2.3	5.5	8.1	10.1	1.2	3.4
Miscellaneous, %	3.5	3.3	8.1	11.7	4.1	4.1
-OPD, %	7.6	5.8	7.0	6.8	4.2	6.9

*SSRI* selective serotonin reuptake inhibitors, *SSNRI* serotonin-norepinephrine reuptake inhibitors, *TCA* tricyclic antidepressants, Miscellaneous antidepressants include bupropion, vilazodone, maprotiline, mirtazapine, nefazodone and trazodone. -OPD: excluding antidepressants that are also approved for other psychiatric disorders: SSRIs escitalopram, fluoxetine, fluvoxamine, paroxetine, sertraline, and SSNRI venlafaxine

Table 3 characterized the general impact on health and life according to the severity of depressive symptoms based on PHQ-9 scores. Among people with T2DM who had moderately severe or severe depressive symptoms (PHQ-9 score ≥ 15), 23.2 % reported that these problems made it extremely difficult for them to do their work, take care of things at home, or get along with people. In contrast, less than 1 % of those with none or minimal depressive symptoms (PHQ-9 score 0–4) reported such negative impact. Further, about 1 in 5 of the subjects with moderately severe or severe depressive symptoms reported having suicidal thoughts nearly every day. Well over half of the people with moderately severe or severe depressive symptoms reported poor mental health and poor physical health over half of the past 30 days. With respect to combined impact of poor mental and poor physical health, 55.3 % of people with moderately severe or severe depressive symptoms reported that poor physical or poor mental health kept them from usual activities such as self-care, work, school or recreation in more than half of the past 30 days. About half of the people with moderate or severe depressive symptoms reported taking antidepressants, but only 1 in 7 reported having seen or talked to a mental health professional such as a psychologist, psychiatrist, psychiatric nurse, or clinical social worker about their health during the past 12 months.

**Fig. 2:**
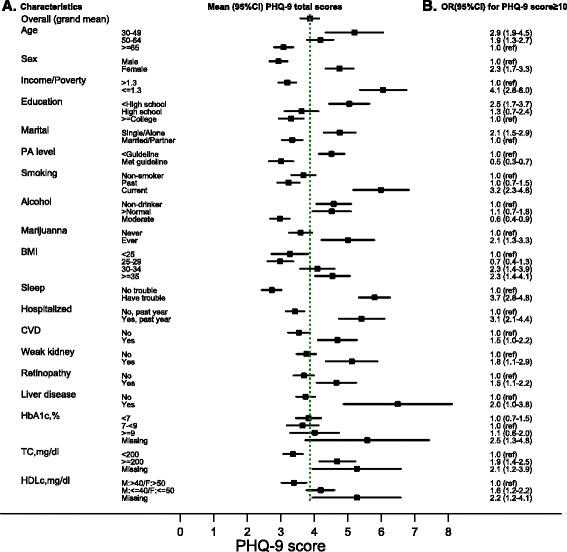
Mean (95 % CI) of PHQ-9 score (panel **a**) and crude ORs for CRD (panel **b**). Legends: squares mark out mean estimates while horizontal lines spread 95 % confidence interval, the green vertical dotted-line indicates overall grand mean of PHQ-9 score (as a continuous variable) for all T2DM, not stratified by any specific characteristics. Panel **a** shows the mean (95 % CI) of continuous PHQ-9 score according to various characteristics in T2DM, Panel **b** shows corresponding crude odds ratio (OR) for clinically relevant depression (CRD), defined by PHQ-9 ≥ 10. CVD, cardiovascular diseases; HDLc, high-density lipoprotein cholesterol; TC, total cholesterol. BMI, body mass index; PA, physical activity. While PHQ-9 score ranges from 0 to 27, x-axis is not drawn to full 27 points to save space

Figure 2 plots the mean PHQ-9 scores for statistically significant variables in the univariate analysis that were associated with CRD (i.e., PHQ-9 score ≥ 10), and corresponding crude odds ratio (OR) estimates from the survey logistic regression models. Overall, the mean PHQ-9 total score in people with T2DM was 3.9 (95 % CI 3.6–4.1). The OR estimates are consistent with descriptions in Table 1, for example, people with T2DM in the age category of 30–49 were almost 3-fold as likely to have CRD as those aged 65 years or older, with OR = 2.9 (95 % CI 1.9–4.5). Female sex is associated with an OR of 2.3 (95 % CI 1.7–3.3) for CRD. Lower income (family income less than 130 % of poverty level) is associated with an OR of 4.1 (95 % CI 2.8–6.0) for CRD. Body mass index greater than 30 (i.e., obesity) or 35 (i.e., severe obesity) is associated with CRD with ORs about 2.

We also evaluated uni-variable associations with CSD (i.e., PHQ-9 score ≥ 15); variables that achieved statistical significance were a subset of those identified for CRD above. The mean PHQ-9 scores and the OR estimates for these variables were also plotted (Additional file 4) and overall, the OR estimates for the variables in association with CSD appeared larger than the corresponding estimates for CRD.

Table 4 presents results of multi-variable regression models that only retained statistically significant predictors for CRD and CSD. Significant predictors of CRD include age, gender, income, education, smoking, body mass index, sleep problems, hospitalization in the past 12 months, and total cholesterol not at goal (≥200 mg/dl). Significant predictors of CSD include age, gender, income, education, physical activity, sleep problems, cardiovascular diseases, and total cholesterol not at goal (≥200 mg/dl).

**Table 4:** ORs (95 % CIs) of statistically significant predictors for CRD and CSD in the multivariable model

Variable/characteristic	Category	CRD (PHQ-9 depression total scores ≥ 10)	CSD (PHQ-9 depression total scores ≥ 15)
Age, years	30–49	2.0 (1.3–3.3)	3.3 (1.5–7.0)
	50–64	1.5 (1.0–2.2)	3.3 (2.0–5.5)
	≥65	1.0 (ref.)	1.0 (ref.)
Sex	Male	1.0 (ref.)	1.0 (ref.)
	Female	2.1 (1.4–3.1)	1.8 (1.0–2.9)
Family income/poverty ratio	> 1.3	1.0 (ref.)	1.0 (ref.)
	≤ 1.3	2.8 (1.8–4.4)	3.1 (1.6–5.8)
Education level	Below high school	2.0 (1.3–3.1)	3.1 (1.7–5.6)
	High school graduate	1.3 (0.7–2.4)	1.5 (0.7–3.0)
	College or above	1.0 (ref.)	1.0 (ref.)
Physical Activity level^a^	Below guideline	–	1.0 (ref.)
	Met guideline	–	0.4 (0.2–0.7)
Smoking status	Non-smoker	1.0 (ref.)	1.0 (ref.)
	Former smoker	1.3 (0.9–2.0)	–
	Current smoker	2.4 (1.7–3.6)	–
Body Mass Index, kg/m^2^	< 25	1.0 (ref.)	–
	25–29	0.9 (0.4–1.8)	–
	30–34	3.0 (1.6–5.5)	–
	≥ 35	2.4 (1.3–4.6)	–
Sleeping status	No trouble	1.0 (ref.)	1.0 (ref.)
	Have trouble	3.0 (2.1–4.2)	3.7 (2.4–5.7)
Hospitalized in the past 12 months	No	1.0 (ref.)	–
	Yes	2.6 (1.8–3.7)	–
Cardiovascular diseases	No	–	1.0 (ref.)
	Yes	–	2.2 (1.3–3.8)
Total cholesterol, mg/dL	< 200	1.0 (ref.)	1.0 (ref.)
	≥ 200	1.7 (1.2–2.4)	2.0 (1.2–3.6)

*CI* confidence interval, *OR* odds ratio, *ref*. reference; ^a^Physical activity level evaluated according to the 2008 Physical Activity Guidelines for Americans [42]

## Discussion

To the best of our knowledge, this study is the first to provide a US national level estimates of depression prevalence for people with T2DM [8, 9].

We estimated an overall point prevalence of 10.6 % (95 % CI 8.9–12.2) for clinically relevant depression (CRD, PHQ-9 score ≥ 10) in people with T2DM, which is about 1.56 times that of the general US adult population (6.8 % using NHANES data 2005–2008) [27]. Similarly, our overall prevalence estimate of 4.2 % for clinically *significant* depression (CSD, PHQ-9 score ≥ 15) in people with T2DM is about 1.75 times that of the general US adult population (2.4 %) [27]. Interestingly, our study found that 49.4 % of the T2DM people with CSD reported taking antidepressants, which is about 1.55 times of the 31.8 % reported for the general US adult population with CSD [27]. This suggests that people with T2DM who had CSD are more likely to receive antidepressant medications than in the general population of the US with CSD. This could be due to the higher rate of interaction of subjects with T2DM with health care providers. Likewise, when we consider antidepressant medication in combination with CRD, the burden of depressive symptoms on people with T2DM overall may be about 25.4 %, and in women it may be as high as about 32 % (Fig. 1).

A previous meta-analysis based on studies before 2005 has reported depression prevalence of 17.6 % among people with T2DM, which appeared higher than our estimated prevalence for CRD and CSD, but lower than our estimate of the burden of depression combined with antidepressant treatment. This may suggest that recognition and treatment of depression has improved for people with T2DM, even though some people may be taking the antidepressant medications for symptoms or diseases other than depression, or perhaps physicians may prescribe more antidepressants to people with chronic diseases. For example, duloxetine is a selective serotonin and norepinephrine reuptake inhibitor (SNRI) antidepressant that is also indicated for neuropathic pain for people with T2DM. However, only 0.8 % of people with T2DM reported taking duloxetine (Table 3). In a sensitivity analysis that excluded antidepressants approved for other psychiatric disorders (mainly anxiety and related disorders, see Table 3, last row), our estimated overall prevalence of CRD and/or antidepressants was 16.7 % (95 % CI 14.6–18.9 %) in people with T2DM. These estimates are likely too conservative (i.e., underestimation of the prevalent burden of depression among people with T2DM) because of (1) the assumption that none of the excluded common antidepressants were taken for depressive symptoms, or none affected PHQ-9 scores at all, and (2) the common comorbidity of depression and other psychiatric conditions, particularly anxiety disorders. For example, one Canadian community-based study of about 2,000 people with T2DM [28] found 74 % (135 of 183) of people with above-threshold anxiety symptoms (defined as score ≥ 10 on the 7-item Generalized Anxiety Disorder Questionnaire (GAD-7)) also had comorbid depression symptoms (defined as PHQ-9 score ≥ 10). We conducted extensive literature search but couldn’t find data on the prevalence of comorbid depression and anxiety among people with diabetes in the US, consistent with the observation made by the Canadian study that “despite this frequently observed co-morbidity, there is little research that explicitly looks at both anxiety and depression in people with diabetes.” Even in the general US population, we only found one publication that provided data (in their Supplemental Table S3) for estimating the prevalence of co-morbid depression among people with anxiety [29]: among participants in the Detroit Neighborhood Health Study, 2008–2010, 78.9 % of those with anxiety (defined as GAD-7 score 15–21) also had depression (defined as modified PHQ-9 with cutoff 3+). The prevalence of (self-reported physician diagnosed) diabetes was 18.3 % among a total of 1,050 participants in that study. Furthermore, the prevalence of PHQ-9 score ≥10, and/or having seen a mental health professional in the past 12 months, and/or taking antidepressants (excluding the 6 antidepressants with multiple indications) was 22.7 % (95 % CI 20.0–25.4 %) (Additional file 3). Additional analyses showed consistent positive correlation between taking antidepressants and higher PHQ-9 scores (Additional file 5).

Depression by itself has been associated with reduced quality of life, loss of work days, damage to relationships, and even suicide [30, 31]. Our study has found such negative impact to be particularly concentrated among people with moderately severe or severe depressive symptoms (PHQ-9 score ≥ 15), and antidepressant treatment alone may not have achieved remission of depressive symptoms for them [32]. Although NHANES is a large, nationally representative survey that provides comprehensive data, the cross-sectional data does not provide longitudinal follow-up, and effectiveness of depression medications cannot be evaluated.

Cross-sectional studies cannot clarify whether patients are depressed because they have disabling and worrisome diabetes complications, or whether having depression could actually precede occurrence of diabetes complications [33]. The biological mechanisms by which depression and type 2 diabetes are associated remain unclear [34]. In addition to a growing body of literature indicating a bidirectional association between these two serious long-term diseases [35], shared biological and behavioral pathways that may simultaneously predispose to both disorders have been proposed [36].

Depression prevalence estimates may also depend on the clinical and methodological settings, such as clinical versus community, self-reported questionnaires versus standardized diagnostic interviews, etc. [8]. Used most often in primary care settings, the PHQ-9 has been shown to be a reliable and valid tool for screening depression [37], including in the elderly [38], and has been applied in studies of diabetes [39]. In fact, some primary care practices were able to integrate PHQ-9 questionnaire with electronic health records (EHR), and such enriched medical records have helped provide higher quality care for depression [40]. Even sub-clinical depression is associated with worse self-care behavior, and may affect all aspects of diabetes treatment [5]. We have identified some easily measured significant predictors for CRD and CSD, which may help with targeted depression screening. Results of stratified analysis by common comorbidities in T2DM (regardless of statistical significance) are given in additional file 6. The management of recognized depression in people with T2DM mainly involves psychotherapy, antidepressant medications, and collaborative care that commonly employs treatment steps or algorithms that include both some psychotherapy and antidepressant medications [41].

Missing data must also be considered in the prevalence estimates. For example, if people with severe depression are less likely to complete the PHQ-9 questionnaire, it could lead to underestimation of the prevalence of severe depression symptoms. We examined the missing data pattern, including individual questions on PHQ-9 questionnaire, but did not detect any notable patterns in demographic and general characteristics between people with missing PHQ-9 data and people with complete data. The most common pattern of missing data is missing all 9 questions.

## Conclusions

The prevalence of clinically *relevant* depression (CRD) and clinically *significant* depression (CSD) among people with T2DM in the US may be lower than in earlier studies, however, the burden of depression remains high. Psychological consultation is less than 15 % (Table 3) even for people with clinically *significant* depression. All health care providers should be aware of mental-health comorbidities while treating a the physical condition of patients with T2DM. Significant predictors of both CRD and CSD include younger age, female sex, lower education level, lower family income, and trouble sleeping. Specific predictors of CRD also include smoking, body mass index ≥30 kg/m^2^, and hospitalization in the past 12 months; specific predictors of CSD included physical activity below recommended level, cardiovascular diseases, and high total cholesterol.

Further research with longitudinal follow-up for depression in people with T2DM is needed to understand real world effectiveness of depression management.

### Ethics approval and consent to participate

The present study was exempt of ethics approval as a secondary analysis of existing NHANES pubic data under the US Health & Human Services (HHS)’ regulations at 45 CFR 46.101(b)(4) (available from http://www.hhs.gov/ohrp/policy/cdebiol.html, last accessed December 2014). US National Center for Healthcare Statistics Research Ethics Review Board (ERB) approved the NHANES surveys (http://www.cdc.gov/nchs/nhanes/irba98.htm, last accessed March 15, 2016). Adult participants gave written informed consent both before home interview and before the exams. Consent forms are available from http://www.cdc.gov/nchs/nhanes.htm.

### Consent for publication

Not applicable.

### Availability of data and materials

All NHANES public-use survey data and related documents are freely available from http://www.cdc.gov/nchs/nhanes/nhanes_questionnaires.htm, with additional web-links provided for each 2-year survey cycle. Additional files 1, 2, 3, 4, 5 and 6 mentioned in the main text are available as Supplemental Materials.

## Additional files

Additional file 1: Title “Response rate in NHANES”, provides survey response rate by each NHANES survey cycle. (DOCX 28 kb) Additional file 2: Title “Characteristics of People With T2DM by PHQ-9 Depression Scores, NHANES 2005–2012”, description according to 4 categories of PHQ-9 score. (DOCX 40 kb) Additional file 3: Title “PHQ-9 Score and Depression Prevalence Estimates in T2DM, by NHANES survey cycles”, analysis by each survey cycle. (DOCX 30 kb) Additional file 4: Title “Mean (95 % CI) of PHQ-9 score (panel A) and crude ORs for CSD (panel B).”, results for CSD. (DOCX 37 kb) Additional file 5: Title “Odds ratio (95 % confidence interval) for taking antidepressants (yes vs no) comparing different PHQ-9 score cutoffs \among people with T2DM”, analysis of positive correlation between PHQ-9 score and antidepressants. (DOCX 28 kb) Additional file 6: Title “Stratified analysis according to comorbidities”, analysis by comorbidities regardless of statistical significance. (DOCX 29 kb)

## Abbreviations

BMI: body mass index
CRD: clinically relevant depression
CSD: clinically significant depression
CVD: cardiovascular diseases
HDLc: high-density lipoprotein cholesterol
MEC: mobile examination center
NHANES: the National Health and Nutrition Survey
PA: physical activity
PHQ-9: Patient Health Questionnaire, 9
TC: total cholesterol
US: United States
T2DM: type 2 diabetes mellitus
