# Supplementary material for: Depression among people with type 2 diabetes mellitus, US National Health and Nutrition Examination Survey (NHANES), 2005–2012
Source: BMC Psychiatry. 2016 Apr 5;16:88. doi: 10.1186/s12888-016-0800-2 (PMC4820858; doi:10.1186/s12888-016-0800-2)
Supplement: Additional file 1: — Title “Response rate in NHANES”, provides survey response rate by each NHANES survey cycle. (DOCX 28 kb) [file 12888_2016_800_MOESM1_ESM.docx]

## Additional file 1. Response rate in NHANES (<http://www.cdc.gov/nchs/nhanes/response_rates_CPS.htm>, last accessed March 15, 2016)

| **Survey Cycle** | **# individuals sampled** | **# of individuals interviewed** | **Response rate, %** |
| --- | --- | --- | --- |
| *1999-2000* | *12,160* | *9,965* | *81.9* |
| *2001-2002* | *13,156* | *11,039* | *83.9* |
| *2003-2004* | *12,761* | *10,122* | *79.3* |
| 2005-2006 | 12,862 | 10,348 | 80.5 |
| 2007-2008 | 12,943 | 10,149 | 78.4 |
| 2009-2010 | 13,272 | 10,537 | 79.4 |
| 2011-2012 | 13,431 | 9,756 | 72.6 |

Interview response rate overall was about 80% from between 2005 and 2010, but 73% in the 2011-2012 cycle. According to the National Center for Health Statistics (<http://www.cdc.gov/nchs/data/nhanes/analytic_guidelines_11_12.pdf>, last accessed March 15, 2016 ), nonresponse bias analysis was conducted for the 2011-2012 survey cycle. Results from the nonresponse bias analysis showed that “very few estimates indicated large relative differences across the stages of weighting for interviewed persons, and none indicated large relative differences for examined persons”, However, several of the characteristics found to be significantly related to response status were either used or highly correlated with those used in the weighting adjustments indicating that the bias may have been reduced through the weighting adjustments. ” Design-based analytic procedures explicitly take into account features of the survey design such as differential selection probabilities and geographic clustering (<http://www.cdc.gov/nchs/data/series/sr_01/sr01_056.pdf>, last accessed March 15, 2016)
