# Supplementary material for: Depression among people with type 2 diabetes mellitus, US National Health and Nutrition Examination Survey (NHANES), 2005–2012
Source: BMC Psychiatry. 2016 Apr 5;16:88. doi: 10.1186/s12888-016-0800-2 (PMC4820858; doi:10.1186/s12888-016-0800-2)
Supplement: Additional file 2: — Title “Characteristics of People With T2DM by PHQ-9 Depression Scores, NHANES 2005–2012”, description according to 4 categories of PHQ-9 score. (DOCX 40 kb) [file 12888_2016_800_MOESM2_ESM.docx]

## Additional file 2. Characteristics of People With T2DM by PHQ-9 Depression Scores, NHANES 2005–2012

| **Characteristic** | **PHQ-9 depression score** | | | | | Overall |
| --- | --- | --- | --- | --- | --- | --- |
|  | **0–4** | **5–9** | **10–14** | **15–27** | Missing |  |
|  | **None/minimal** | Mild | Moderate | **Moderately severe/ Severe** |  |  |
| N, participants (%) | 1,322 (60.6) | 369 (16.9) | 147 (6.7) | 109 (5.0) | 235 (10.8) | 2,182 (100) |
| Frequency, weighted ^a^ (%) | 9,512,027 ^a^ (62.9) | 2,578,599 ^a^  (17.1) | 959,837 ^a^ (6.3) | 637,638 ^a^  (4.2) | 1,429,515 ^a^  (9.5) | 15,117,616 ^a^ (100) |
| Age group, %  30–49 years  50–64 years  65–74 years  ≥ 75 years | 14.4  37.3  28.9  19.4 | 20.6  39.6  24.2  15.6 | 29.0  39.4  21.0  10.7 | 25.4  50.3  15.4  8.9 | 12.1  40.7  16.8  30.4 | 16.6  38.7  25.8  18.8 |
| Sex, %  Male  Female | 55.3  44.7 | 35.1  64.9 | 31.6  68.4 | 29.4  70.6 | 50.3  49.7 | 48.7  51.3 |
| Race, %  Non-Hispanic white  Non-Hispanic black  Mexican and other Hispanic  Other | 64.1  17.4  11.7  6.8 | 59.4  16.3  15.0  9.2 | 57.3  20.5  18.6  3.7 | 50.6  23.4  21.8  4.2 | 44.3  18.6  16.8  20.3 | 60.4  17.8  13.6  8.2 |
| Family income/poverty ratio<=1.3, % | 18.6 | 24.7 | 44.9 | 62.9 | 28.7 | 24.1 |
| Marital status, single or living alone, % | 32.8 | 42.3 | 55.3 | 53.3 | 49.2 | 38.3 |
| Education,% |  |  |  |  |  |  |
| College or above | 49.8 | 44.3 | 36.9 | 23.7 | 35.1 | 45.5 |
| High school graduate | 26.4 | 25.5 | 24.6 | 19.5 | 18.0 | 25.0 |
| Below high school | 23.9 | 30.1 | 38.5 | 56.8 | 46.9 | 29.4 |
| Smoking,%  Non-smoker  Past  Current | 49.9  38.8  11.3 | 51.6  29.5  18.9 | 39.4  31.0  29.6 | 39.1  24.7  36.1 | 57.4  29.0  13.6 | 49.8  35.2  15.0 |
| Alcohol,%  Non-drinker  Above moderate level  Moderate drinking | 34.1  16.1  49.8 | 46.8  23.7  29.5 | 43.6  21.5  34.9 | 58.9  20.1  21.0 | 89.4  2.5  8.1 | 43.1  16.6  40.3 |
| Marijuana, ever use, % | 17.3 | 22.9 | 28.6 | 29.3 | Missing | 17.8 |
| Physical activity level met guidelines, ^b^ % | 46.7 | 40.5 | 33.2 | 17.2 | 27.3 | 41.7 |
| Cardiovascular diseases | 25.0 | 33.6 | 32.7 | 41.9 | 33.8 | 28.5 |
| Diabetic retinopathy | 17.0 | 19.6 | 30.0 | 18.9 | 21.9 | 18.8 |
| Hypertension | 83.5 | 80.1 | 83.1 | 79.3 | 71.9 | 81.6 |
| Liver diseases | 3.2 | 8.4 | 5.1 | 12.1 | 3.0 | 4.5 |
| Cancer | 18.2 | 15.7 | 14.9 | 23.8 | 15.7 | 17.5 |
| Weak/failing kidneys, %^c^ | 7.1 | 7.2 | 11.8 | 13.2 | 8.0 | 7.8 |
| My Health in general is,%  Excellent, very good  Good/Fair  Poor  Missing | 20.9  74.1  5.0  0.0 | 7.1  80.0  12.8  0.0 | 3.3  67.4  29.2  0.0 | 1.2  61.7  37.1  0.0 | 1.9  20.4  4.8  72.9 | 14.8  69.1  9.2  6.9 |
| Hours of sleep per day, %  <=5 hours  6-8 hours  >=9 hours  Missing | 26.2  64.4  8.6  0.8 | 35.1  57.7  6.1  1.1 | 43.5  40.2  15.6  0.7 | 23.8  61.4  12.4  2.4 | 18.6  71.6  9.2  0.6 | 26.2  64.4  8.6  0.8 |
| Self-reported having trouble sleep,% | 28.5 | 51.1 | 62.6 | 69.2 | 37.6 | 37.1 |
| Told by doctor having sleep disorders,% | 16.0 | 14.5 | 24.4 | 38.2 | 15.8 | 17.2 |
| Age at diabetes diagnosis, years, mean (s.e.)  Categories,%  <50  50-69  >=70  Missing | 52.8 (0.4)  40.6  48.8  10.1  0.5 | 51.3 (0.8)  46.2  44.4  8.7  0.7 | 49.2 (1.1)  55.2  39.1  4.8  0.9 | 48.3 (1.3)  56.1  37.2  4.8  1.9 | 52.1 (0.9)  39.9  47.6  10.1  2.4 | 52.1 (0.4)  43.0  46.8  9.3  0.9 |
| Duration of diabetes, mean (s.e.)  Categories,%  <5 years  5-9 years  >=10 years  Missing | 9.8 (0.3)  31.1  24.5  43.9  0.5 | 9.7 (0.5)  34.7  21.3  43.2  0.8 | 8.4 (0.7)  33.0  30.2  35.9  0.9 | 9.6 (1.0)  21.1  29.3  47.6  1.9 | 11.6 (0.7)  21.9  24.9  50.8  2.4 | 9.9 (0.2)  30.5  24.5  44.1  0.8 |
| HbA1c, %, Mean (s.e.)  Categories,%  <6.5%  6.5-<7.5%  7.5-8.9%  >=9%  Missing | 7.2 (0.1)  35.0  29.1  19.7  12.6  3.7 | 7.2 (0.1)  40.2  29.3  15.2  12.6  2.7 | 7.4 (0.2)  35.5  23.0  21.9  12.4  7.3 | 7.4 (0.2)  31.7  28.7  11.9  15.0  12.8 | 7.5 (0.1)  23.8  29.3  20.3  16.6  10.0 | 7.3 (0.1)  34.7  28.7  18.8  13.0  4.7 |
| Total cholesterol (mg/dl)  Mean (s.e.)  Categories,%  >0 and <200  >=200  Missing | 178.8 (1.8)  68.5  26.2  5.3 | 192.9 (3.5)  59.4  33.5  7.1 | 191.8 (5.2)  54.8  37.6  7.6 | 195.3 (5.3)  42.4  43.4  14.2 | 183.6 (4.7)  59.1  29.0  11.9 | 183.1 (1.5)  64.1  29.2  6.7 |
| LDL cholesterol(mg/dl), mean (s.e.)  Categories,%  >0 and <100  >=100  Missing | 96.0 (1.7)  55.6  37.0  7.4 | 103.7 (3.5)  43.5  46.3  10.2 | 105.6 (5.4)  48.8  44.8  6.5 | 112.9 (6.0)  38.9  54.3  6.8 | 102.4 (5.0)  49.7  43.2  7.1 | 99.0 (1.4)  52.0  40.2  7.8 |
| HDL cholesterol (mg/dl)  Male and female, mean (s.e.)  Male, mean (s.e.)  Female, mean (s.e.)  Categories,%  Male>40 and female>50  Male<=40 and female<=50  missing | 47.8 (0.5)  44.4 (0.6)  52.1 (0.9)  50.7  44.0  5.3 | 47.5 (0.9)  42.4 (1.1)  50.2 (1.0)  47.3  45.7  7.1 | 46.3 (1.4)  42.6 (2.9)  48.1 (1.6)  40.3  52.0  7.6 | 45.1 (1.7)  38.7 (2.7)  48.0 (1.6)  31.7  54.0  14.2 | 48.5 (1.1)  44.8 (1.4)  52.3 (1.9)  47.6  40.5  11.9 | 47.6 (0.4)  44.0 (0.5)  51.2 (0.7)  48.4  44.9  6.7 |
| Triglycerides (mg/dl)  Mean (s.e.)  Categories,%  >0 and <150  >=150  missing | 174.7 (12.3)  56.5  41.3  2.2 | 192.7 (18.3)  51.7  44.5  3.8 | 190.7 (18.3)  45.5  53.5  1.0 | 162.4 (14.7)  47.6  45.5  6.8 | 156.7 (13.4)  57.0  40.8  2.2 | 176.6 (9.4)  54.8  42.6  2.6 |
| Blood pressure (BP), mmHg  SBP, mean (s.e.)  DBP, mean (s.e)  Categories, %  SBP<140, DBP<90  Missing | 130.6 (0.7)  68.3 (0.6)  68.3  3.0 | 130.6 (1.4)  69.0 (0.7)  67.8  3.2 | 129.9 (1.9)  71.9 (1.3)  77.4  1.0 | 126.6 (2.1)  68.8 (1.9)  64.3  4.5 | 130.5 (1.9)  67.8 (1.1)  57.2  21.0 | 130.4 (0.5)  68.6 (0.4)  67.5  4.6 |
| Medications |  |  |  |  |  |  |
| Insulin,% | 27.1 | 25.2 | 21.0 | 30.3 | 29.6 | 26.8 |
| Sulfonylureas,% | 36.1 | 32.8 | 30.3 | 22.1 | 39.0 | 34.8 |
| THIAZOLIDINEDIONES,% | 18.4 | 11.6 | 17.3 | 9.9 | 21.6 | 17.1 |
| Meglitinides,% | 2.0 | 2.1 | 0.0 | 0.4 | 1.2 | 1.8 |
| DPP-4 INHIBITORS,% | 7.1 | 9.0 | 2.3 | 0.0 | 8.2 | 6.9 |
| GLP-1 AGONISTS,% | 1.8 | 0.9 | 1.1 | 2.9 | 0.4 | 1.5 |
| Metformin,% | 54.6 | 48.2 | 63.7 | 53.7 | 43.7 | 53.0 |
| Any antihyperglycemic agents | 88.3 | 85.8 | 91.1 | 81.6 | 91.0 | 88.0 |
| Antihypertensives, % | 78.1 | 77.7 | 77.9 | 73.4 | 65.0 | 76.6 |
| Statins,% | 56.4 | 47.0 | 56.5 | 45.2 | 53.7 | 53.9 |
| Body mass index, kg/m^2^, mean (s.e.)  Categories,%  <25  25-<30  30-<35  35+  Missing | 32.5 (0.3)  12.9  27.0  29.1  30.9  0.1 | 33.4 (0.4)  13.4  24.6  22.5  39.3  0.1 | 36.3 (0.8)  7.2  9.4  37.9  45.6  0.0 | 34.4 (0.7)  9.6  13.5  35.9  41.0  0.0 | 31.0 (0.7)  21.6  27.9  25.2  25.3  0.0 | 32.8 (0.2)  13.3  25.0  28.5  33.2  0.1 |
| Waist-to-height ratio, mean (s.e.) | 0.656 (0.004) | 0.673 (0.006) | 0.704 (0.008) | 0.690 (0.010) | 0.647 (0.009) | 0.663 (0.003) |
| Categories,% |  |  |  |  |  |  |
| 0.3-<0.5 | 2.4 | 2.9 | 1.1 | 1.8 | 1.2 | 2.3 |
| 0.5-<0.7 | 67.0 | 55.9 | 49.6 | 46.5 | 45.5 | 61.1 |
| ≥0.7 | 26.7 | 33.4 | 42.1 | 37.4 | 15.0 | 28.2 |
| Missing | 3.9 | 7.8 | 7.2 | 14.3 | 38.3 | 8.5 |

Values are % unless stated otherwise, percentages may not add up exactly to 100 due to rounding.

^a^ There were 466 participants (=2,648-2,182) who reported having diagnosed diabetes but were not included in this analysis (due to presumed type 1 diabetes or non-participation in the MEC exams), representing 3,653,851 (=18,771,467-15,117,616) people with diabetes in the US civilian, non-institutionalized population.

^b^ Physical activity level evaluated according to the 2008 Physical Activity Guidelines for Americans[42].

^c^A “yes” answer to “*Have you ever been told by a doctor or other health professional that you have weak or failing kidneys (excluding kidney stones, bladder infections, or incontinence)?*”
