# Supplementary material for: Depression among people with type 2 diabetes mellitus, US National Health and Nutrition Examination Survey (NHANES), 2005–2012
Source: BMC Psychiatry. 2016 Apr 5;16:88. doi: 10.1186/s12888-016-0800-2 (PMC4820858; doi:10.1186/s12888-016-0800-2)
Supplement: Additional file 3: — Title “PHQ-9 Score and Depression Prevalence Estimates in T2DM, by NHANES survey cycles”, analysis by each survey cycle. (DOCX 30 kb) [file 12888_2016_800_MOESM3_ESM.docx]

## Additional file 3. PHQ-9 Score and Depression Prevalence Estimates in T2DM, by NHANES survey cycles

| Survey Cycle | 2005-06 | 2007-08 | 2009-10 | 2011-12 | Overall |
| --- | --- | --- | --- | --- | --- |
| N, T2DM | 401 | 603 | 606 | 572 | 2182 |
| %,taking Antidepressants | 21.7 (16.4-27.0) | 17.2 (11.4-22.9) | 19.2 (14.3-24.1) | 20.5 (16.2-24.8) | 19.6 (17.3-22.0) |
| %, -OPD | 5.5 (3.0-8.0) | 6.2 (3.7-8.7) | 10.1(6.5-13.7) | 5.6 (1.9-9.4) | 6.9 (5.3-8.4) |
| % -OPD or seen mental health professional | 12.0 (7.7-16.3) | 12.5 (9.2-15.8) | 18.4 (14.2-22.6) | 12.7 (5.8-19.5) | 13.9 (11.5-16.3) |
| PHQ-9 score ≥10 or –OPD, or seen mental health professional | 17.6 (13.3-21.9) | 23.2 (18.2-28.3) | 27.6 (23.0-32.1) | 22.0 (14.5-29.4) | 22.7 (20.0-25.4) |
| % seen mental health professional, and no antidepressants | 6.5 (3.3-9.8) | 5.8 (3.6-8.0) | 8.9 (5.8-12.0) | 8.5 (2.1-15.0) | 7.5 (5.5-9.6) |
| % missing PHQ-9 score | 10.4 (7.4-13.3) | 9.8 (6.5-13.2) | 11.0 (8.4-13.6) | 7.1 (4.3-9.8) | 9.5 ( 8.1-10.8) |
| % missing PHQ-9 score and not taking antidepressants | 7.9 (4.9-11.0) | 8.4 (4.9-11.9) | 9.4 (6.6-12.1) | 5.6 (3.6-7.5) | 7.7 ( 6.4- 9.1) |
| % taking antidepressants missing PHQ-9 data | 2.4 (0.9-3.9) | 1.4 (0.2-2.7) | 1.6 (0.8-2.4) | 1.5 (0.1-2.9) | 1.7 ( 1.1- 2.3) |
| %,PHQ-9 score≥10 | 6.7 (4.3-9.1) | 11.6 (7.2-16.0) | 11.8 (8.6-15.0) | 11.7 (8.1-15.3) | 10.6 ( 8.9-12.2) |
| %,PHQ-9 score≥15 | 1.7 (0.4-3.0) | 3.2 (2.2-4.2) | 5.6 (4.0-7.3) | 5.8 (3.1-8.5) | 4.2 ( 3.4- 5.1) |
| %,PHQ-9 score≥15 not taking antidepressants | 0.7 (0.1-1.4) | 1.4 (0.6-2.1) | 3.1 (1.7-4.5) | 3.0 (1.3-4.8) | 2.1 ( 1.5- 2.8) |
| %,PHQ-9 score≥20 | 0.9 (0.0-2.1) | 1.1 (0.3-1.9) | 1.5 (0.3-2.8) | 1.8 (0.1-3.5) | 1.4 ( 0.7- 2.0) |
| %,PHQ-9 score≥20 not taking antidepressants | 0.1 (0.0-0.4) | 0.6 (0.1-1.0) | 0.8 (0.1-1.8) | 1.3 (0.3-3.0) | 0.8 ( 0.2- 1.3) |
| %, PHQ-9 score≥10, or taking antidepressants | 25.6 (19.8-31.4) | 24.1 (19.0-29.2) | 25.6 (19.9-31.2) | 26.3 (21.9-30.8) | 25.4 (23.0-27.9) |
| %, PHQ-9 score≥15, or taking antidepressants | 22.4 (17.5-27.4) | 18.5 (12.9-24.2) | 22.3 (16.5-28.1) | 23.5 (19.2-27.9) | 21.8 (19.3-24.2) |
| %, taking antidepressants and PHQ-9 score<5 or missing | 15.3 (11.3-19.3) | 8.3 ( 4.4-12.3) | 10.2 ( 6.9-13.6) | 9.2 ( 5.7-12.7) | 10.6 ( 8.9-12.3) |
| %, taking antidepressants and PHQ-9 score <5, not missing | 12.9 ( 8.7-17.1) | 6.9 ( 3.8-10.1) | 8.6 ( 5.2-12.1) | 7.7 ( 4.6-10.8) | 8.9 ( 7.3-10.5) |
| %, taking antidepressants, and PHQ-9 score 5-9 | 3.6 ( 1.1- 6.2) | 4.2 ( 1.2- 7.2) | 3.5 ( 1.2- 5.8) | 5.4 ( 2.3- 8.6) | 4.3 ( 2.9- 5.6) |
| %, taking antidepressants and PHQ-9 score 10-14 | 1.8 ( 0.0- 3.6) | 2.8 ( 0.8- 4.8) | 2.9 ( 0.9- 4.9) | 3.1 ( 1.2- 4.9) | 2.7 ( 1.8- 3.6) |
| %, taking antidepressants and PHQ-9 score 15-27 | 1.0 (-0.2- 2.1) | 1.8 ( 0.9- 2.8) | 2.5 ( 1.3- 3.8) | 2.8 ( 0.9- 4.7) | 2.1 ( 1.4- 2.7) |
| %, taking antidepressants and PHQ-9 score <10 or missing | 18.9 (13.7-24.2) | 12.5 ( 6.9-18.2) | 13.8 ( 9.8-17.8) | 14.6 (10.2-19.0) | 14.9 (12.6-17.1) |
| %, taking antidepressants and PHQ-9 score <10, not missing | 16.5 (11.1-22.0) | 11.1 ( 5.9-16.3) | 12.2 ( 8.1-16.2) | 13.1 ( 9.4-16.8) | 13.2 (11.0-15.3) |
| %, taking antidepressants and PHQ-9 score <15 or missing | 20.7 (15.4-26.1) | 15.3 ( 9.7-21.0) | 16.7 (11.8-21.6) | 17.7 (13.4-22.0) | 17.5 (15.2-19.9) |
| %, taking antidepressants and PHQ-9 score <15 not missing | 18.3 (12.8-23.8) | 13.9 ( 8.9-19.0) | 15.1 (10.1-20.1) | 16.2 (12.5-19.8) | 15.8 (13.6-18.1) |

*note, roughly about 8.7% (i.e., 1.7/19.6) of those taking antidepressants had missing PHQ-9, and about 9.6% (i.e., 7.7/80.4) of those not taking antidepressants had missing PHQ-9, so no evidence that those on antidepressants were more likely to miss PHD-9 data. -OPD: excluding antidepressants that are also approved for other psychiatric disorders: SSRIs escitalopram, fluoxetine, fluvoxamine, paroxetine, sertraline, and SSNRI venlafaxine.
