# Supplementary material for: Depression among people with type 2 diabetes mellitus, US National Health and Nutrition Examination Survey (NHANES), 2005–2012
Source: BMC Psychiatry. 2016 Apr 5;16:88. doi: 10.1186/s12888-016-0800-2 (PMC4820858; doi:10.1186/s12888-016-0800-2)
Supplement: Additional file 4: — Title “Mean (95 % CI) of PHQ-9 score (panel A) and crude ORs for CSD (panel B).”, results for CSD. (DOCX 37 kb) [file 12888_2016_800_MOESM4_ESM.docx]

## Additional file 4. Mean (95% CI) of PHQ-9 score (panel A) and crude ORs for CSD (panel B)

1. **Characteristics Mean (95%CI) PHQ-9 total scores B. OR(95%CI) for PHQ-9 score≥15**

Marker legends: squares mark out mean estimates while horizontal lines spread 95% confidence interval, the green vertical dotted line indicates overall grand mean PHQ-9 score for all T2DM, not stratified by any specific characteristics. Panel A shows the mean (95% CI) of continuous PHQ-9 score according to various characteristics in T2DM, Panel B shows corresponding crude odds ratio (OR) for clinically relevant depression (CRD), defined by PHQ-9≥10. CVD, cardiovascular diseases; HDLc, high-density lipoprotein cholesterol; TC, total cholesterol. BMI, body mass index; PA, physical activity. While PHQ-9 score ranges from 0 to 27, x-axis is not drawn to full 27 points to save space.
