# Supplementary material for: Depression among people with type 2 diabetes mellitus, US National Health and Nutrition Examination Survey (NHANES), 2005–2012
Source: BMC Psychiatry. 2016 Apr 5;16:88. doi: 10.1186/s12888-016-0800-2 (PMC4820858; doi:10.1186/s12888-016-0800-2)
Supplement: Additional file 5: — Title “Odds ratio (95 % confidence interval) for taking antidepressants (yes vs no) comparing different PHQ-9 score cutoffs \among people with T2DM”, analysis of positive correlation between PHQ-9 score and antidepressants. (DOCX 28 kb) [file 12888_2016_800_MOESM5_ESM.docx]

## Additional file 5. Odds ratio (95% confidence interval) for taking antidepressants (yes vs no) comparing different PHQ-9 score cutoffs among people with T2DM

| Comparing PHQ-9 score | Restricted to those with | Crude model* | Additionally adjusted for covariates** |
| --- | --- | --- | --- |
| >=15 vs <15 (ref) | - | 4.35 (2.70-7.03) | 2.47 (1.50-4.06) |
| >=10 vs <10 (ref) | - | 4.11 (2.98-5.68) | 2.44 (1.62-3.65) |
| >=10 vs <10 (ref) | PHQ score<15 | 3.66 (2.43-5.52) | 2.27 (1.38-3.73) |
| >=5 vs <5 (ref) | - | 2.82 (2.13-3.73) | 1.77 (1.25-2.51) |
| >=5 vs <5 (ref) | PHQ score<15 | 2.45(1.80-3.33) | 1.65 (1.15-2.36) |
| >=5 vs <5 (ref) | PHQ score<10 | 1.93 (1.32-2.84) | 1.39 (0.90-2.15) |

*Crude model adjusted for survey design only (stratification, clustering, and nonresponse)

**Adjusted for all the covariates selected in the final models for CRD and CSD (see Table 4 of main text)

Overall, higher depressive symptom scores are associated with current use of antidepressants, the association appeared stronger among people with higher PHQ-9 scores. Although correlation between PHQ-9 scores and antidepressant use (correlation coefficient about 0.3) can be estimated from the cross-sectional survey, the interpretation may be confounded by underlying depression severity and duration as well as (hopefully) treatment effectiveness of the antidepressants. Some people taking antidepressants with PHQ-9 score<10 may have been effectively managed by the antidepressants, while others taking antidepressants with higher PHQ-9 scores may have already improved symptoms but have yet to achieve better control.
