# Supplementary material for: Depression among people with type 2 diabetes mellitus, US National Health and Nutrition Examination Survey (NHANES), 2005–2012
Source: BMC Psychiatry. 2016 Apr 5;16:88. doi: 10.1186/s12888-016-0800-2 (PMC4820858; doi:10.1186/s12888-016-0800-2)
Supplement: Additional file 6: — Title “Stratified analysis according to comorbidities”, analysis by comorbidities regardless of statistical significance. (DOCX 29 kb) [file 12888_2016_800_MOESM6_ESM.docx]

## Additional file 6. Stratified analysis according to comorbidities

|  | **OR (95% CI) for** | | **Prevalence (%) of** | | | |
| --- | --- | --- | --- | --- | --- | --- |
|  | PHQ-9 score≥10 | PHQ-9 score≥15 | PHQ-9 score≥10 | PHQ-9 score≥15 | PHQ-9 score≥10 and/or antidepressants | PHQ-9 score≥15 and/or antidepressants |
| Cardiovascular diseases |  |  |  |  |  |  |
| Yes | 1.2 (0.8-1.8) | 2.2 (1.3-4.0) | 13.3 ( 9.9-16.8) | 6.1 ( 4.3- 8.0) | 33.3 (28.8-37.8) | 29.6 (25.1-34.1) |
| No | 1 (reference) | 1 (reference) | 9.4 ( 7.5-11.4) | 3.4 ( 2.4- 4.4) | 22.2 (19.2-25.3) | 18.6 (15.8-21.4) |
| Diabetic retinopathy |  |  |  |  |  |  |
| Yes | 1.3 (0.9-1.9) | 0.8 (0.4-1.6) | 14.3 (10.5-18.0) | 4.2 ( 2.1- 6.3) | 26.0 (20.6-31.3) | 20.8 (15.9-25.7) |
| No | 1 (reference) | 1 (reference) | 9.7 ( 7.8-11.6) | 4.2 ( 3.1- 5.4) | 25.3 (22.3-28.3) | 22.0 (19.1-24.9) |
| Hypertension |  |  |  |  |  |  |
| Yes | 1.1 (0.7-1.7) | 0.8 (0.4-1.5) | 10.5 ( 8.8-12.3) | 4.1 ( 3.1- 5.1) | 26.1 (23.5-28.7) | 22.4 (19.7-25.2) |
| No | 1 (reference) | 1 (reference) | 10.7 ( 7.3-14.1) | 4.8 ( 2.6- 7.1) | 22.5 (17.3-27.6) | 18.7 (13.8-23.7) |
| Liver diseases |  |  |  |  |  |  |
| Yes | 1.4 (0.7-2.8) | 2.5 (1.0-6.3) | 18.2 ( 9.4-27.0) | 11.2 ( 3.3-19.1) | 43.9 (30.2-57.6) | 41.5 (27.7-55.2) |
| No | 1 (reference) | 1 (reference) | 10.2 ( 8.4-12.0) | 3.9 ( 3.0- 4.8) | 24.6 (22.0-27.1) | 20.8 (18.4-23.3) |
| Cancer |  |  |  |  |  |  |
| Yes | 1.4 (0.9-2.2) | 1.9 (1.1-3.2) | 11.1 ( 7.4-14.9) | 5.7 ( 2.7- 8.7) | 24.2 (18.2-30.1) | 19.7 (13.8-25.6) |
| No | 1 (reference) | 1 (reference) | 10.4 ( 8.7-12.2) | 3.9 ( 3.0- 4.8) | 25.7 (23.1-28.3) | 22.2 (19.7-24.7) |
| Weak/failing kidneys |  |  |  |  |  |  |
| Yes | 1.2 (0.6-2.2) | 1.1 (0.5-2.3) | 16.6 (10.7-22.4) | 7.1 ( 3.8-10.4) | 36.6 (28.5-44.7) | 30.8 (21.9-39.6) |
| No | 1 (reference) | 1 (reference) | 10.1 ( 8.2-11.9) | 4.0 ( 3.0- 5.0) | 24.5 (22.1-26.9) | 21.0 (18.7-23.3) |

CI, confidence interval; OR, prevalence odds ratio.
